# Emergence of Epidemic Dengue-1 Virus in the Southern Province of Sri Lanka

**DOI:** 10.1371/journal.pntd.0004995

**Published:** 2016-10-06

**Authors:** Champica K. Bodinayake, L. Gayani Tillekeratne, Ajith Nagahawatte, Vasantha Devasiri, Wasantha Kodikara Arachichi, John J. Strouse, October M. Sessions, Ruvini Kurukulasooriya, Anna Uehara, Shiqin Howe, Xin Mei Ong, Sharon Tan, Angelia Chow, Praveen Tummalapalli, Aruna D. De Silva, Truls Østbye, Christopher W. Woods, Duane J. Gubler, Megan E. Reller

**Affiliations:** 1 Department of Medicine, Faculty of Medicine, University of Ruhuna, Galle, Sri Lanka; 2 Division of Infectious Diseases, Department of Medicine, Duke University School of Medicine, Durham, North Carolina, United States of America; 3 Duke Global Health Institute, Durham, North Carolina, United States of America; 4 Department of Microbiology, Faculty of Medicine, University of Ruhuna, Galle, Sri Lanka; 5 Department of Pediatrics, Faculty of Medicine, University of Ruhuna, Galle, Sri Lanka; 6 Teaching Hospital Karapitiya, Galle, Sri Lanka; 7 Department of Pediatrics, Johns Hopkins University School of Medicine, Baltimore, Maryland, United States of America; 8 Emerging Infectious Diseases Programme, Duke NUS Graduate Medical School, Singapore; 9 Duke Ruhuna Collaborative Research Center, Faculty of Medicine, University of Ruhuna, Galle, Sri Lanka; 10 Genetech Research Institute, Colombo, Sri Lanka; 11 Department of Community and Family Medicine, Duke University School of Medicine, Durham, North Carolina, United States of America; 12 Hubert-Yeargan Center for Global Health, Durham, North Carolina, United States of America; 13 Division of Infectious Diseases, Department of Medicine, Johns Hopkins University School of Medicine, Baltimore, Maryland, United States of America; Institute for Disease Modeling, UNITED STATES

## Abstract

**Background:**

Dengue is a frequent cause of acute febrile illness with an expanding global distribution. Since the 1960s, dengue in Sri Lanka has been documented primarily along the heavily urbanized western coast with periodic shifting of serotypes. Outbreaks from 2005–2008 were attributed to a new clade of DENV-3 and more recently to a newly introduced genotype of DENV-1. In 2007, we conducted etiologic surveillance of acute febrile illness in the Southern Province and confirmed dengue in only 6.3% of febrile patients, with no cases of DENV-1 identified. To re-evaluate the importance of dengue as an etiology of acute febrile illness in this region, we renewed fever surveillance in the Southern Province to newly identify and characterize dengue.

**Methodology/Principal Findings:**

A cross-sectional surveillance study was conducted at the largest tertiary care hospital in the Southern Province from 2012–2013. A total of 976 patients hospitalized with acute undifferentiated fever were enrolled, with 64.3% male and 31.4% children. Convalescent blood samples were collected from 877 (89.6%). Dengue virus isolation, dengue RT-PCR, and paired IgG ELISA were performed. Acute dengue was confirmed as the etiology for 388 (39.8%) of 976 hospitalizations, with most cases (291, 75.0%) confirmed virologically and by multiple methods. Among 351 cases of virologically confirmed dengue, 320 (91.2%) were due to DENV-1. Acute dengue was associated with self-reported rural residence, travel, and months having greatest rainfall. Sequencing of selected dengue viruses revealed that sequences were most closely related to those described from China and Southeast Asia, not nearby India.

**Conclusions/Significance:**

We describe the first epidemic of DENV-1 in the Southern Province of Sri Lanka in a population known to be susceptible to this serotype because of prior study. Dengue accounted for 40% of acute febrile illnesses in the current study. The emergence of DENV-1 as the foremost serotype in this densely populated but agrarian population highlights the changing epidemiology of dengue and the need for continued surveillance and prevention.

## Author Summary

Dengue is a globally emerging cause of fever that is caused by four serotypes of virus (DENV1-4). Since the 1960s, dengue in Sri Lanka has been documented primarily along the heavily urbanized western coast. Outbreaks from 2005–2008 were attributed to a new type of DENV-3 and more recently to a newly introduced type of DENV-1. Having identified serotypes other than DENV-1 as an important cause of fever in southern Sri Lanka in 2007, our prospective surveillance in 2012 enabled detection and characterization of an epidemic of dengue that caused 388 (39.8%) hospitalizations for fever. DENV-1 accounted for 91.2% of 351 virologically confirmed cases; isolates were most closely related to sequences described from China and Southeast Asia, not nearby India. Acute dengue was associated with self-reported rural residence, travel, and rainfall. Emergence of DENV-1 as the foremost serotype in the densely populated but agrarian southern Sri Lanka highlights the changing epidemiology of dengue and need for continued surveillance and prevention.

## Introduction

Dengue virus (DENV), a frequent cause of acute febrile illness with an expanding global distribution, is a flavivirus with 4 antigenically distinct serotypes (DENV-1–4) that is transmitted by *Aedes aegypti* and *Aedes albopictus* mosquitoes [1]. Infection with a single serotype leads to long-term protective immunity against the homologous serotype but not against other serotypes [2]. In the past decades, urbanization, globalization, and lack of control of *Aedes aegypti* have contributed to the global spread of the disease [3]. It is now estimated that 50% of the world’s population live in areas where dengue virus has the potential to be transmitted [4].

Dengue was first serologically confirmed in Sri Lanka in 1962 and the initial outbreak was recorded in 1965 [5]. Although all four serotypes were known to be co-circulating in the country, dengue hemorrhagic fever/ dengue shock syndrome (DHF/DSS) was rare in Sri Lanka before 1989 [6]. From 1989 through 2008, epidemics with periodic shifting of serotypes and increasing magnitude and severity occurred every few years, with the later epidemics being associated with a new clade of DENV-3. However, since 2009, there has been a dramatic increase in dengue transmission with an average of 33,000 cases reported per year. The predominant virus associated with these recent outbreaks has been a newly introduced genotype of DENV-1 [7].

In 2007, our research team conducted etiologic surveillance of acute febrile illness at the largest tertiary care hospital in the Southern Province. Dengue was confirmed in only 6.3% of febrile patients seeking acute care at the hospital and no cases of DENV-1 were identified [8]. To re-evaluate the importance of dengue as an etiology of acute febrile illness in the Southern Province, we renewed fever surveillance at this large tertiary care hospital in 2012. We report here the epidemiologic features of an epidemic of DENV-1 that occurred during our latter surveillance period.

## Methods

### Febrile cohort

We conducted a cross-sectional surveillance study for febrile illness from June 2012 through May 2013 in the adult and pediatric wards of Teaching Hospital Karapitiya, the largest (1,500 bed) tertiary care hospital in the Southern Province of Sri Lanka. This hospital provides both primary and tertiary care to a catchment area of approximately 1 million people in the Galle District. The hospital also serves as the primary teaching hospital for the Southern Province (total population 2.5 million) and receives referrals from other hospitals in the province [9]. Consecutive patients ≥1 year of age with documented fever at presentation or within 48 hours of hospital admission were eligible for enrollment. We excluded patients who presented with focal bacterial infections, such as pneumonia or soft tissue infection.

MBBS-qualified study physicians recorded epidemiologic and clinical data and trained phlebotomists collected an acute blood sample at the time of enrollment. Patients returned 2–4 weeks after enrollment for a convalescent blood sample or were visited at home if their address was known. Serum samples were stored promptly at -80°C and shipped on dry ice to the Duke-NUS Graduate Medical School in Singapore for laboratory testing.

### Laboratory testing

#### IgG ELISA

Paired sera for each patient were tested on the same ELISA plate with an in-house IgG ELISA as previously described [8, 10]. Plates were coated overnight at 4°C with purified DENV-2 antigen at 117.6 ng/ML in coating buffer (pH 9.6). After overnight incubation, plates were washed five times with PBST (1X PBS+0.05%Tween 20), blocked with 1% NFDM in PBS at room temperature for 1 hour, and washed five times with PBST in an automatic plate washer. Patient sera were diluted 1:100 in PBS and 50μl were added to each well. The plates were incubated at 37°C for 1hr and washed five times with PBST. Goat anti-human IgG HRP antibody diluted 1:1000 in blocking buffer was added (25μl) to each well, incubated at 37°C for 1hr, washed five times with PBST, and blotted dry. TMB (100μl) was added to each well and the plates were incubated for seven minutes at room temperature after which 50μl of stop solution were added. Plates were read at 450nm on a plate reader.

#### Dengue virus isolation

Individual aliquots of acute-phase serum were diluted 1:20 with L-15 maintenance medium and inoculated on to a monolayer of C6/36 *Aedes albopictus* cells. Cells were incubated at 30°C with 5% CO_2_ for 10 days and observed daily for cytopathic effects (CPE). At 10 days (or earlier if CPE was obvious), cells were fixed and an indirect immunofluorescence assay was performed using mouse monoclonal antibodies to DENV1-4 and anti-mouse antibodies conjugated to FITC. Samples were subjected to at least 2 passages before being deemed positive or negative.

#### RT-PCR for DENV

RNA was isolated from aliquots of acute phase serum using the Qiagen RNeasy Mini kit. cDNA synthesis and quantitative RT-PCR were performed using the Invitrogen SuperScript III Platinum One–Step Quantitative RT PCR System. Primers and probes used to amplify and differentially detect dengue serotypes were developed by the Centers for Disease Control and Prevention, Atlanta, Biotech Core Facility as described previously [11].

#### Sequence analysis

Full genome sequencing of dengue virus isolates was done in accordance with the protocols described in Christenbury et al. [12]. Sanger sequencing reads were processed and assembled into full genomes with the Geneious 8.1.7 program. The full genome sequences for these samples have been deposited into GenBank (KT445955, KT445956, KT445957, KT445958, KT445959, KT445960, KT445961, KT445962, and KT445963). The nine full genomes from the present study were then aligned with 1604 unique, full genome DENV1 sequences present in GenBank using MAFFT. FastTree 2.1.5 was then used to infer an approximately-maximum-likelihood phylogenetic tree. The clade consisting of the 9 Galle sequences and 15 other sequences (KP 398852, KJ468234, KJ726664, KJ726662, KJ726663, JN054255, HQ891313, HQ891314, JN054256, HQ891316, HQ891315, EU280167, FJ196844, FJ176779, and AB608787) was selected. 25 other sequences representative of the tree’s diversity were selected by taking sequences (GU131678, JQ922548, FV537256, HI553375, KF921911, GU131973, AB074760, JQ287661, JQ287664, JQ287663, GU131778, JF459993, JQ287662, KF955446, KJ649286, KJ189363, AB204803, GU131833, JQ915076, AB519681, HG316481, AY835999, KF921935, KF921933, and EU863650) from different clades of the tree. These sequences were then aligned using MAFFT and a maximum likelihood tree constructed using raxmlGUI 1.5 beta with a general-time reversible GAMMA nucleotide model and ML+Rapid Bootstrapping algorithm with 1000 bootstrap replicates.

### Laboratory definitions

We required definitive serologic evidence (IgG seroconversion), definitive virologic evidence (positive PCR and either isolation or positive PCR with a second target), or both serologic and virologic evidence (positive convalescent IgG and either positive PCR or isolation) to confirm acute dengue. Acute primary (first episode) and acute secondary (recurrent) dengue were distinguished by the absence or presence of IgG in acute-phase serum samples, respectively, as defined previously [8]. Those with inconclusive acute dengue (virologic evidence of dengue or flavivirus infection but not confirmed) were excluded. Patients with positive IgG in both acute and convalescent sera but without evidence of acute infection were classified as past dengue. Seroprevalence was defined as the presence of IgG in acute-phase serum samples, as we have done previously [8].

### Epidemiologic and statistical analysis

The proportions of patients who met criteria for confirmed acute dengue were calculated. Associations between patients’ sociodemographic characteristics and dengue infection were evaluated. We compared epidemiological characteristics of patients with confirmed acute dengue versus no acute dengue. Categorical variables were compared using the Chi square test or Fisher exact test and continuous variables were compared using the t-test or Kruskall-Wallis test. We performed multivariable logistic regression to identify characteristics associated with acute dengue versus no dengue, adjusting for sex and age as a continuous variable. Rainfall data for the Galle region during the study months were obtained from the Sri Lanka Department of Meteorology, Colombo [13]. STATA, version 11 (STATACorp, College Station, Texas) was used for all statistical analyses.

### Ethics statement

Written informed consent was obtained from patients or their guardians (for patients <18 years of age) and written assent was obtained from patients aged 12–17 years. The institutional review boards of Ruhuna University (Sri Lanka), Johns Hopkins University, and Duke University Medical Center approved the study.

## Results

### Febrile cohort

We enrolled 976 patients and obtained convalescent samples from 877 (89.6%). A total of 628 (64.3%) were male and 306 (31.4%) were children < 18 years. The median duration of hospitalization was 5 (IQR 4–6) days. Six (0.6%) patients were transferred to an intensive care unit and 4 (0.4%) died in hospital. The median day of illness the acute sample was drawn was 4 days (IQR 3–6), and the median time between acute-phase and convalescent follow up was 23 days (IQR 16–39). The likelihood of follow-up was greater in children than in adults (94.8% versus 87.6%, p<0.001), but did not differ by sex (p = 0.61), level of education (p = 0.15), or reported urban vs. rural residence (p = 0.86). The reported duration of fever (p = 0.41) and illness (p = 0.73) was similar in patients who did and did not return for follow-up.

### Acute dengue

Overall, acute dengue was confirmed as the etiology for 388 (39.8%) of the 976 hospitalizations for acute febrile illness during the study period. Acute dengue was either confirmed or excluded in 937 patients, since 39 had inconclusive evidence of acute dengue, Of the 388 with acute dengue, 100 were confirmed by IgG seroconversion. Most were virologically confirmed and by multiple methods, since 291 were confirmed by dengue PCR with a positive convalescent IgG, 161 by viral isolation with a positive convalescent IgG, 166 were both virus isolation and dengue-specific PCR positive, and 136 were dengue PCR and flavivirus PCR-positive. Few (15) virologically confirmed cases of acute dengue were viral isolation-positive but dengue PCR-negative; of these. 5 were flavivirus PCR-positive and the remainder had supportive serology (convalescent IgG positive). The mean reported duration of illness was shorter (4.0 d versus 5.2 d, p = 0.006) among those confirmed by isolation and PCR versus than among those confirmed by seroconversion alone.

**Fig 1:**
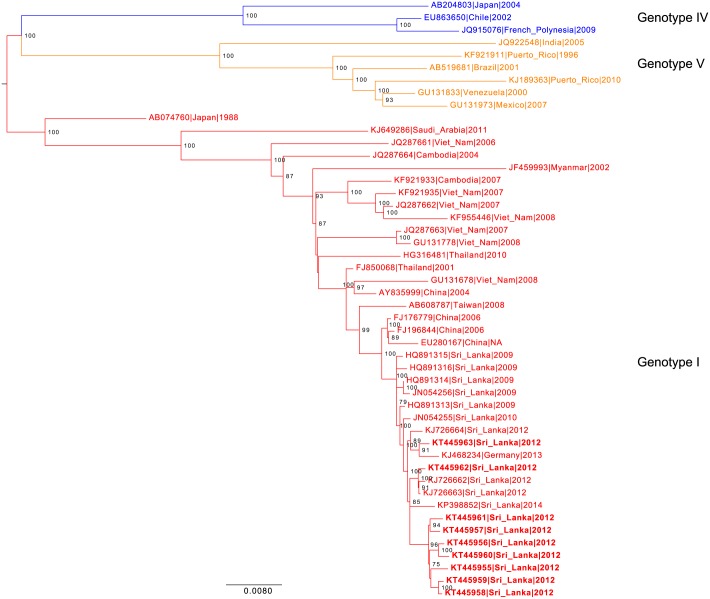
Phylogenetic analysis of DENV-1 isolates from Galle, Sri Lanka between 2012–2013. DENV-1 nucleotide sequences from Sri Lanka were aligned with representative DENV-1 sequences from around the world representing multiple genotypes. Blue-colored isolates represent genotype IV, red-colored isolates represent genotype I and orange-colored isolates represent genotype V. The DENV-1 sequences from this study (KT445955, KT445956, KT445957, KT445958, KT445959, KT445960, KT445961, KT445962, and KT445963) are depicted in bold.

Among 351 patients with laboratory-confirmed acute dengue who were dengue PCR or virus isolation-positive, 320 (91.2%) were DENV-1, 25 (7.1%) were DENV-4, and 6 (1.7%) were DENV-2. A dendrogram of representative isolates of DENV-1 is shown in Fig 1. Among the 388 patients with acute dengue, 103 (26.5%) had primary dengue, 245 (63.1%) had secondary dengue, and 40 (10.3%) could not be classified because insufficient acute sample was available to test for IgG.

### Sociodemographic characteristics of those with and without acute dengue infection

**Fig 2:**
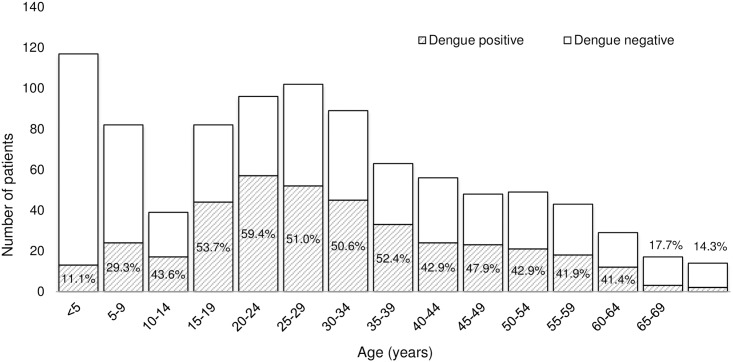
Number and proportion of patients admitted with acute dengue as cause of acute febrile illness by age group at a tertiary care hospital in southern Sri Lanka, June 2012- May 2013.

Among 937 patients in whom acute dengue was either confirmed or excluded (39 with inconclusive acute dengue removed from analysis dataset), 606 (64.7%) were male and 645 (68.8%) were adults. The median age of enrolled patients was 27.2 years (IQR 14.3–42.2). Dengue accounted for over half of febrile illnesses in adolescents and young adults (those 15 to 39 years of age); the proportion of acute febrile illness attributable to acute dengue in each age group is depicted in Fig 2. Sociodemographic characteristics of those with acute dengue versus no acute dengue are compared in Table 1. Reported residence in a rural area (38.7% versus 24.4%), a history of travel in the previous 30 days (27.9% versus 17.2%), and residing further from the hospital (25km versus 18km) were more common in those with acute dengue than in those without acute dengue in bivariable analyses (p<0.001 for all) as well as in analyses adjusted for age and sex (Table 1).

**Table 1:** Demographic characteristics of febrile patients with acute dengue or no evidence of acute dengue, n = 937, Southern Sri Lanka, 2012–13.

Sociodemographic and clinical characteristics^	Acute dengue^(n = 388)	No dengue^(n = 549)	Adjusted OR (95% CI)	Adjusted p-value^+^
**Age, years**	28.3 (19.5–41.5)	26.1 (6.8–43.1)	—	—
**Male**	250 (64.4)	356 (64.9%)	—	—
**Rural residence**	150 (38.7)	134 (24.4%)	1.94 (1.46–2.58)	**<0.001**
**Travel past 30d**	107 (27.9)	94 (17.2%)	1.84 (1.34–2.53)	**<0.001**
**Residence (km) from hospital**	25 (10.0–50.0)	18.0 (8.0–30.0)	1.01 (1.00–1.01)	**0.001**
**Education** ≥12^th^ grade*	118 (30.6)	110 (20.3)	1.68 (1.24–2.27)	**<0.001**
Occupation*				0.18
Unemployed/ retired	69 (23.9)	94 (29.2)	1.12 (0.95–1.33)	
Laborer	99 (34.3)	126 (39.1)		
Merchant/office	52 (18.0)	45 (14.0)		
Other	69 (23.9)	57 (17.7)		
**Days in hospital**	5 (4–7)	4 (3–6)	1.01 (0.99–1.02)	0.33
**Died**	0 (0)	4 (0.7)	—	0.98

* if ≥18 years;

^ Proportions (%) except median (IQR) for age and distance. IQR, interquartile range.

^+^Adjusted for age and sex.

### Seroprevalence of dengue

**Fig 3:**
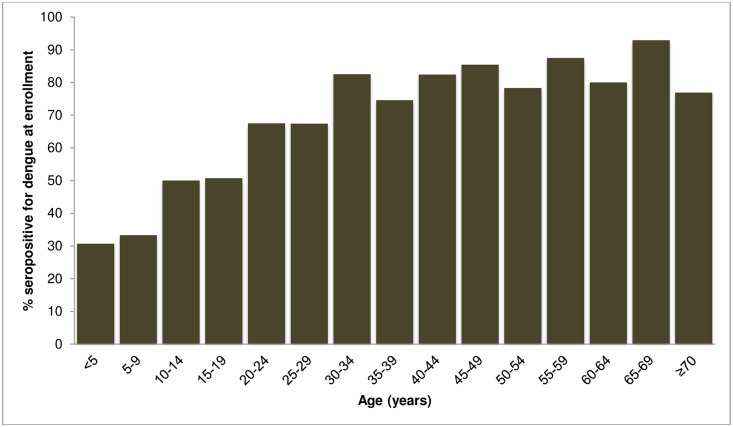
Proportion seropositive for dengue by age group, southern Sri Lanka, July 2012- May 2013.

A total of 255 patients without confirmed acute dengue had laboratory results consistent with past dengue. The proportion of patients who were seropositive at enrollment increased with age from 30.7% in those <5 years old to 85.4% in those 45–49 years old (Fig 3). Presence of IgG at enrollment was similar in male and female patients (56.9% versus 54.1%, respectively) and in rural and urban dwellers (54.9% versus 56.4%, respectively).

### Monthly variation in acute dengue and rainfall

**Fig 4:**
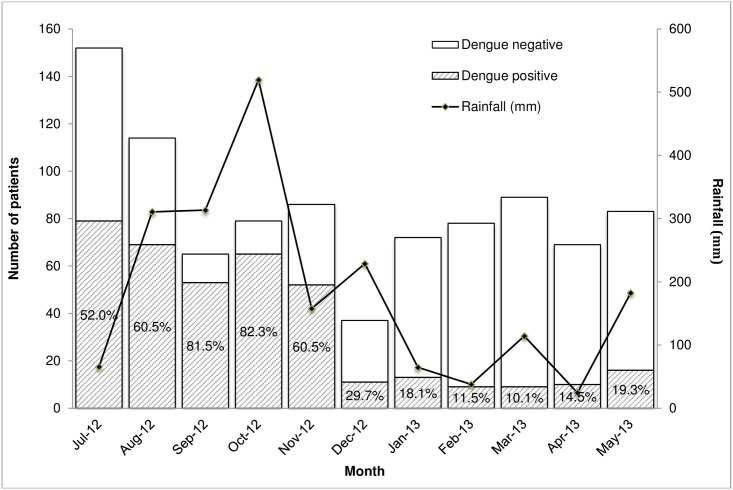
Proportion with acute dengue among patients hospitalized with acute febrile illness at a tertiary care hospital in southern Sri Lanka, June 2012- May 2013. Monthly rainfall for the Galle district is depicted by the line graph.

Acute dengue occurred during each month of the study, with a clear increase associated with the seasonal monsoons. Acute dengue accounted for the largest proportion of febrile illness cases in October 2012 (82.3%), which was also the month with the greatest rainfall during our study period (519.5mm). Monthly variation in proportion of acute dengue and monthly rainfall for the Galle district are depicted in Fig 4.

### Sequence analysis

Dengue virus isolates from this Galle cohort were closely related to each other as well as to viruses isolated from other areas in Sri Lanka. The other viruses from Sri Lanka were all isolated from patients in the Western Province, with the exception of one for whom the location could not be traced (KP398852). One closely related genotype 1 sequence was from Germany (KJ468234), but was isolated from a peripheral blood stem cell recipient with acute myeloblastic leukemia whose donor had recently contracted acute dengue while traveling in Sri Lanka [14]. The clade containing the Sri Lankan and German sequences also included three sequences from China and one from Taiwan. This clade is most closely related to sequences from China and Southeast Asia, especially Thailand and Vietnam (Fig 1), as has also been noted by others who, during the same period as this study, sequenced isolates from the Western Province and found them also most closely related to those from China [15]. Interestingly, samples from India were more distantly related despite the geographical proximity of Sri Lanka to India and primacy as trade partners (https://atlas.media.mit.edu/en/profile/country/lka/).

## Discussion

By repeating acute febrile illness surveillance at the largest tertiary care center in the Southern Province of Sri Lanka, we were uniquely positioned to describe the changing epidemiology of dengue at this hospital, which serves a large proportion of the region’s population. Comparing our study in 2012 with our previous study in 2007, we detected an increase in acute dengue as a cause of AFI (from 6.3% to 40%), a shift in serotype to DENV-1 in a susceptible population (no DENV-1 identified in 2007), and a new association of dengue with reported rural rather than urban residence. In short, in agrarian but still densely populated southern Sri Lanka, we identified dengue 1 as the cause of epidemic dengue associated with reported rural rather than urban residence. Expanded virologic testing and the high proportion of patients in whom virologic confirmation was achieved (dengue virus isolated or PCR-positive or both) provided conclusive evidence for DENV-1 as the cause of the dengue epidemic. This is the first report to describe epidemic DENV-1 in the Southern Province of Sri Lanka.

The high proportion with acute dengue in 2012 versus 2007 (39.8% vs. 6.3%) and seroprevalence in 2012 similar to that observed in the Western Province in 2012 rather than that observed in 2007 are consistent with an island-wide dengue epidemic that began in 2009 [16]. In 2012, 44,461 cases of dengue were reported to the Sri Lankan Ministry of Health from throughout the country. Although this represents the largest number of cases of dengue reported to date in a single year in Sri Lanka, the majority of cases (70%) were only clinically diagnosed. In contrast, we used rigorous diagnostic methods to conservatively but conclusively document the magnitude of the DENV-1 epidemic at this hospital, which provides both primary and tertiary care for a large proportion of patients residing in the region [17]. We assert that the population studied is representative of the general population in the area, since only 6% of hospital admissions during 2012–2013 were due to transfers from other hospitals. Additionally, care at government run Teaching Hospital Karapitiya is free for all. Finally, although we enrolled only those hospitalized in this study, 92.6% of those with illness subsequently confirmed as acute dengue were hospitalized in our prior study [8]. This is in accord with Sri Lankan guidelines for management of dengue, wherein those with platelet counts of less than or equal to 100,000 are hospitalized.

In our study, dengue accounted for at least 10% of acute febrile illness during each month of the study period, with a high of 82% in October 2012 and a low of 10% in March 2013. Notably, October 2012 was the month with the greatest rainfall during our study period (519.5mm) whereas March 2013 had relatively less rainfall (114.1mm) [13]. The association between dengue and rainfall has been previously described, since rainfall produces conditions that are favorable for reproduction and survival of the vector mosquitoes [18, 19]. Further surveillance studies must be continued in this region to delineate patterns of disease associated with seasonal and climatic changes such that appropriate prevention measures can be planned and carried out.

Traditionally, dengue has been considered an urban disease, and disease surveillance and prevention efforts have focused on the control of *Aedes aegypti* larval habitats in urban-associated containers used for storing water, used tires, and non-biodegradable plastic and metal packaging for consumer goods [20]. Although dengue has been reported from throughout Sri Lanka, most cases have been recognized in the heavily urbanized Western Province [7, 21–24]. In this study, reported rural residence was surprisingly more common in those with acute dengue than in those with no evidence of acute dengue. There have been increasing reports of dengue in rural areas worldwide, but this pattern of transmission is not yet fully understood [25–28]. Travel to urban centers that have a heavy burden of disease, secondary vectors such as *Aedes albopictus*, and stored water containers because of uncertain water supply have all been implicated as reasons for the increasing prevalence of dengue in rural areas [25]. In our study, a history of recent travel prior to illness was associated with acute dengue. Although we collected information on occupation and travel within 30 days and found these variables to be associated with higher risk of acute dengue infection, we could not determine the exact location where dengue had been acquired. However, in Sri Lanka up to 28% of the labor force in the urban Western Province consists of workers from outside the region who commute for work [29]. Galle is located only 116 km from the urbanized capital of Colombo and is agrarian but still densely populated. As new routes of transportation, such as the Southern Expressway built in 2013, are introduced between the two cities, it is expected that inter-city travel will increase. Further study of patterns of human travel as well as on the characterization of the vector may be important in control efforts.

The vast majority of dengue in our study was caused by DENV-1 serotype, which represents a marked shift from our findings in 2007 when only DENV-2, DENV-3, and DENV-4 were isolated and DENV-3 was the most prevalent [8]. This shift in serotype is similar to what has been observed in the Western Province [6, 7]. Although DENV-1 has been co-circulating in Sri Lanka for decades, large annual epidemics of DENV-1 have only occurred since 2009 with the appearance of a new DENV genotype-1 strain that may have been introduced from China or Thailand [15]. Our phylogenetic analysis revealed that the DENV-1 genotype in southern Sri Lanka is similar to the one that is circulating in the Western Province with both most closely related to those from China, which supports the theory that migration of humans between the two cities may have contributed to the spread of the disease [15]. Our prior surveillance in 2007 suggested that our study population lacked DENV-1 antibodies and was susceptible to the new DENV-1 strain, since dengue antibodies are generally type-specific [2]. In 2007, we found that the dengue seroprevalence reached a plateau of 70% by 40 years of age [8]. In contrast, in this study, nearly 70% were seropositive by age 20 and the seroprevalence reached 90% in those 60–65 years of age.

Strengths of our study include reproducible and objective enrollment criteria (unselected patients with documented fever of defined magnitude) and use of gold standard diagnostic criteria (paired IgG serology as well as isolation and PCR), which was made possible by a high proportion (89.6%) in whom convalescent clinical and serological follow-up was achieved. In 2012 as in 2007 we tested paired sera by IgG ELISA as per Chungue et al. [10], distinguished primary vs. secondary dengue based on the absence or presence of IgG in acute-phase sera, and performed isolation and PCR to serotype dengue. Our estimate of secondary dengue in 2012 is relatively conservative, since we used IgG ELISA as a qualitative test and some proportion of those IgG-positive in both acute and convalescent sera but with negative virologic testing may have had secondary acute dengue. Identification of epidemic dengue 1 at our site in southern Sri Lanka is consistent with reports of epidemic dengue elsewhere in the country by the Sri Lankan Ministry of Health [30].

In conclusion, we describe the first documented epidemic of DENV-1 in the Southern Province of Sri Lanka in a population susceptible to this serotype. Our report expands understanding of the changing epidemiology of acute dengue in the region with acute dengue infections in southern Sri Lanka associated with reported rural (not urban) residence, rainfall, travel, and residence in rural areas (median~ 25km) outside of Galle. Our epidemiologic and phylogenetic analyses highlight the importance of recognized internal travel but also unrecognized international travel, as evidenced by the relatedness of isolates from Sri Lankans to that a German stem cell transplant recipient who had not traveled but his donor had [14]. Our continued surveillance allowed us to detect and describe a marked change in the epidemiology of dengue with the emergence of DENV-1 in this region of southern Sri Lanka. Further characterization of isolated strains and larger population-based epidemiologic studies will be critical to elucidate the geographic expansion of DENV-1, its pathogenicity relative to other serotypes and independent of whether associated with secondary vs primary dengue, and the most effective methods to prevent and control the disease.

## Supporting Information

S1 Checklist STROBE checklist. (DOC)
